# Isolation and Characterization of a Novel Phage SaGU1 that Infects *Staphylococcus aureus* Clinical Isolates from Patients with Atopic Dermatitis

**DOI:** 10.1007/s00284-021-02395-y

**Published:** 2021-02-26

**Authors:** Yuzuki Shimamori, Ajeng K. Pramono, Tomoe Kitao, Tohru Suzuki, Shin-ichi Aizawa, Tomoko Kubori, Hiroki Nagai, Shigeki Takeda, Hiroki Ando

**Affiliations:** 1 Department of Microbiology, Graduate School of Medicine, Gifu University, 1-1 Yanagido, Gifu, Gifu 501-1194 Japan; 2 Division of Molecular Science, Graduate School of Science and Technology, Gunma University, 1-5-1 Tenjin-cho, Kiryu, Gunma 376-8515 Japan; 3 Laboratory of Phage Biologics, Graduate School of Medicine, Gifu University, 1-1 Yanagido, Gifu, Gifu 501-1194 Japan; 4 Genome Microbiology Laboratory, Faculty of Applied Biological Sciences, Gifu University, 1-1 Yanagido, Gifu, Gifu 501-1193 Japan; 5 Prefectural University of Hiroshima, 562 Nanatsuka, Shobara, Hiroshima 727-0023 Japan; 6 G-CHAIN, Gifu University, 1-1 Yanagido, Gifu, Gifu 501-1194 Japan

## Abstract

The bacterium *Staphylococcus aureus*, which colonizes healthy human skin, may cause diseases, such as atopic dermatitis (AD). Treatment for such AD cases involves antibiotic use; however, alternate treatments are preferred owing to the development of antimicrobial resistance. This study aimed to characterize the novel bacteriophage SaGU1 as a potential agent for phage therapy to treat *S. aureus* infections. SaGU1 that infects *S. aureus* strains previously isolated from the skin of patients with AD was screened from sewage samples in Gifu, Japan. Its genome was sequenced and analyzed using bioinformatics tools, and the morphology, lytic activity, stability, and host range of the phage were determined. The SaGU1 genome was 140,909 bp with an average GC content of 30.2%. The viral chromosome contained 225 putative protein-coding genes and four tRNA genes, carrying neither toxic nor antibiotic resistance genes. Electron microscopy analysis revealed that SaGU1 belongs to the *Myoviridae* family. Stability tests showed that SaGU1 was heat-stable under physiological and acidic conditions. Host range testing revealed that SaGU1 can infect a broad range of *S. aureus* clinical isolates present on the skin of AD patients, whereas it did not kill strains of *Staphylococcus epidermidis*, which are symbiotic resident bacteria on human skin. Hence, our data suggest that SaGU1 is a potential candidate for developing a phage therapy to treat AD caused by pathogenic *S. aureus*.

**Supplementary Information:**

The online version contains supplementary material available at 10.1007/s00284-021-02395-y.

## Introduction

*Staphylococcus aureus* is a Gram-positive commensal bacterium present in human microbiota, however, it can also act as an opportunistic pathogen causing several infectious diseases, including pneumonia, endocarditis, bacteremia [1–4], and atopic dermatitis (AD), which is a common inflammatory skin disease that can be caused by abnormal colonization of *S. aureus* [5, 6].

The current treatment for such AD cases includes the use of topical antibiotics; however, the resultant symptomatic improvement is temporary, often resulting in the development of antibiotic resistance [7]. The growth of drug-resistant *S. aureus*, such as methicillin-resistant *S. aureus* (MRSA), has also been reported on the skin of AD patients [8]. In fact, a previous study in the USA, reported that 80% of patients with AD showed colonization of *S. aureus* on the skin, 16% of which were identified as MRSA [9].

Another issue associated with antibiotic treatment of AD treatment is the impact on the commensal bacterial community [10, 11]. *Staphylococcus epidermidis* represents the predominant symbiotic bacterium within the human skin microbiota, the presence of which helps recover the skin barrier function by improving the skin microbiota in patients with AD. Moreover, the lipopeptide produced by *S. epidermidis* enhances the production of antimicrobial peptides on the skin surface of humans and mice; thereby, preventing infection from pathogenic bacteria including *S. aureus* [12–14]. Therefore, an efficient strategy to treat AD that does not affect the human skin microbiota is preferred as an alternative therapy to antibiotic treatment.

Recently, the application of bacteriophages has gained attention as a therapeutic tool for altering the human microbiota [15, 16]. In this study, we isolated a phage SaGU1 that specifically targets *S. aureus* isolated from patients with AD. Here, we describe its genomic information, biophysical stability, and ability to infect previously identified clinical isolates of *S. aureus* and *S. epidermidis* from the skin of patients with AD.

## Materials and Methods

### Bacterial Strains

All bacterial strains used in this study are listed in Table 1. *Staphylococcus* clinical isolates were obtained from Prof. Suzuki’s Laboratory, Gifu University, Gifu, Japan. The bacterial species were confirmed based on the analysis of the V3–V4 region in the 16S rRNA gene [17]. All bacterial strains except *Listeria innocua* KF2492 were grown in lysogeny broth (LB; Formedium, UK) medium at 37 °C. *L. innocua* was grown in brain heart infusion broth (BHI; BD Difco, NJ).

**Table 1:** Genotyping, drug resistance, and phage susceptibility of bacterial strains used in this study

Species	Strain	Source	MLST		Drug resistance	Plaque formation	EOP
			ST	CC			
*Staphylococcus aureus*	1056-1	Clinical isolate from an AD patient	8	CC8	ABPC	+	1.0
	158-F1	Clinical isolate from an AD patient	4	–	–	+	0.8
	107-1	Clinical isolate from an AD patient	15	CC15	ABPC	+	0.8
	159-B1	Clinical isolate from an AD patient	6	CC5	–	+	3.3
	163-R2	Clinical isolate from an AD patient	15	CC15	–	+	1.7
	SA-1	Clinical isolate from an AD patient	8	CC8	ABPC, LVFX	+	0.8
	SA-2	Clinical isolate from an AD patient	6	CC5	ABPC	+	0.3
	SA-3	Clinical isolate from an AD patient	15	CC15	–	+	0.8
	SA-4	Clinical isolate from an AD patient	8	CC8	–	+	1.7
	SA-5	Clinical isolate from an AD patient	398	–	–	+	1.7
	SA-6	Clinical isolate from an AD patient	15	CC15	–	+	0.8
	SA-7	Clinical isolate from an AD patient	8	CC8	ABPC, LVFX	+	0.3
	SA-8	Clinical isolate from an AD patient	25	–	–	–	< 10^−8^
	GTC01187	Clinical isolate, MRSA	5	CC5	MPIPC, ABPC, CEZ, CMZ, CFX	+	0.3
	GTC01196	Clinical isolate, MRSA	239	CC8	MPIPC, ABPC, CMZ, CFX	–	< 10^−8^
	RN4220	Laboratory strain	8	CC8	–	+	0.4
*Staphylococcus epidermidis*	SE-1	Clinical isolate from an AD patient	922	–	ABPC	–	< 10^−8^
	SE-2	Clinical isolate from an AD patient	5	–	–	–	< 10^−8^
	SE-3	Clinical isolate from an AD patient	297	–	ABPC	–	< 10^−8^
	SE-4	Clinical isolate from a healthy person	152	–	–	–	< 10^−8^
	SE-5	Clinical isolate from an AD patient	2	–	MPIPC, ABPC, CEZ, CMZ, IPM, CFX	–	< 10^−8^
	SE-6	Clinical isolate from a healthy person	992	–	–	–	< 10^−8^
	SE-7	Clinical isolate from an AD patient	53	–	–	–	< 10^−8^
*Listeria innocua*	KF2492	Isolate from chicken	ND	ND	ND	–	< 10^−8^
*Salmonella enterica* serovar Typhimurium	LT2	NBRC13245	ND	ND	ND	–	< 10^−8^
*Bacillus subtilis*	NBRC3936	NBRC3936	ND	ND	ND	–	< 10^−8^
*Escherichia coli*	DH5α	Laboratory strain	ND	ND	ND	–	< 10^−8^
*Pseudomonas aeruginosa*	PAO1	Laboratory strain	ND	ND	ND	–	< 10^−8^
	PA14	Laboratory strain	ND	ND	ND	–	< 10^−8^

*MLST* multilocus sequence typing, *ST* sequence type, *CC* clonal complex, *ABPC* amoxicillin, *CEZ* cefazolin sodium salt, *CFX* cefoxitin, *CMZ* cefmetazole sodium salt, *IPM* imipenem, *LVFX* levofloxacin, *MPIPC* Oxacillin sodium, *EOP* efficiency of plating, *ND* not determined

### Multilocus Sequence Typing of *Staphylococcus* Clinical Isolates

Genotyping of *S. aureus* and *S. epidermidis* was performed by multilocus sequence typing (MLST) [18]. Seven housekeeping genes (*arcC*, *aroE*, *glpF*, *gmk*, *pta*, *tpi*, and *yqi* for *S. aureus*, and *arcC*, *aroE*, *gtr*, *mutS*, *pyrR*, *tpiA*, and *yqiL* for *S. epidermidis*) were amplified using PCR and subsequently sequenced (Research Equipment Sharing Promotion Center NGS facility, Gifu University). The sequences were then compared with the published allele profiles of *S. aureus* and *S. epidermidis* available from the PubMLST database (https://pubmlst.org/saureus/).

### Drug Susceptibility Testing

Drug susceptibility was determined according to the Clinical and Laboratory Standards Institute (CLSI) guidelines [19] using a DP32 drug plate (Eiken Chemical, Japan). Briefly, overnight cultures of the selected *Staphylococcus* isolates were diluted to an OD_600_ of approximately 0.25. Next, 25 µL of the diluted bacterial culture was added to 12 mL of LB medium, of which 100 µL was added to each well of the DP32 drug plate. The plate was incubated at 37 °C for 16–20 h. The minimal inhibitory concentrations (MICs) of the strains were determined according to the manufacturer’s instructions and CLSI guidelines. MRSA was defined as *S. aureus* showing an MIC as follows: oxacillin (MPIPC), ≥ 4 μg/mL; cefoxitin (CFX), ≥ 8 μg/mL according to CLSI guidelines.

### Isolation and Propagation of Bacteriophages

Phage screening was performed using sewage samples obtained from the northern plant of the Water and Sewage Division of Gifu City, Gifu, Japan, according to the previously published protocol [20]. Briefly, 1.6 L of sewage samples was centrifuged at 8000×*g* for 20 min at 4 °C, and the resulting supernatant was added to polyethylene glycol 6000 (final concentration, 10% w/v) and NaCl (final concentration, 4% w/v), and stored overnight at 4 °C. The sample was then centrifuged at 10,000×*g* at 4 °C for 90 min, and the resulting precipitate was re-suspended in 2 mL of phage buffer (10 mM Tris–HCl [pH 7.5], 10 mM CaCl_2_, 10 mM MgSO_4_, 70 mM NaCl). After adding a few drops of chloroform, the sample was kept on ice for 6 h. The sample was then centrifuged at 8000×*g* for 10 min, filtrated through 0.22 µm filters (Merck Millipore, Ireland), and stored at 4 °C. Next, 100 µL of the solution and 200 µL of an overnight culture of each *S. aureus* strain were mixed with soft agar, and subsequently overlaid on the LB plate (double-layer plate method). After incubation at 37 °C, the obtained plaques were individually picked up and re-suspended in 100 µL of phage buffer. The titer of the phage lysate was measured by spotting tenfold serially diluted phages on the bacterial lawn and calculated as plaque-forming units (PFU)/mL.

### Transmission Electron Microscopy (TEM)

The phage lysate was prepared for TEM according to a previously published method [21]. Samples were negatively stained with 2% phosphotungstic acid (w/v, pH 7.0) and observed using a JEM-1200EXII electron microscope (JEOL, Japan). Micrographs were taken at an accelerating voltage of 80 kV.

### One-Step Growth Curve

An one-step growth assay was performed according to a previously published method [22]. Bacterial culture (10^6^ colony-forming units (CFU)/mL) of *S. aureus* 1056-1 was mixed with phage lysate at a multiplicity of infection (MOI) of 0.001, and then incubated for 10 min at 37 °C. The mixture was centrifuged at 7000×*g* for 10 min at 4 °C. The supernatant was discarded, and the pellet was washed twice with LB and subsequently re-suspended in an equal volume of LB. Next, the resuspension was incubated at 37 °C with constant agitation at 250 rpm. Samples were collected every 10 min to measure the phage titers. The burst size was calculated by dividing the average titers for the post-burst time points by the average initial titers.

### Thermal and pH Stability Analysis

The thermal stability of SaGU1 was tested by incubating the phage solution in phosphate buffered saline (PBS) without calcium and magnesium [PBS (−)] at 4, 20, 30, 40, 50, 60, 70, and 80 °C for 2 h. Similarly, to assess the stability of phages under acidic and basic conditions, the phages were incubated in PBS (−) at pH 1 to 13 at 37 °C for 2 h. The rate of surviving phages was calculated using the double-layer plate method.

### Phage Host Range

The host range of SaGU1 was determined using the bacterial strains listed in Table 1. Two hundred microliters of stationary phase culture of the host bacteria was mixed with 0.6% soft agar and layered on the LB plate. Next, 2.5 µL of tenfold serially diluted SaGU1 lysate was spotted on a plate in which the host bacteria were overlaid and incubated overnight at 37 °C. The efficiency of plating (EOP) was calculated by dividing the PFU for target bacteria by the PFU for host bacteria.

### Genome Sequencing and Bioinformatics Analysis

The phage genome was extracted as described previously [23]. Genome sequencing was performed using the Illumina MiSeq platform (Illumina, USA) and the MiSeq Reagent Kit v3 (Illumina). DNA libraries were prepared using the Nextera XT DNA Preparation Kit (Illumina) for paired-end analyses. Obtained reads were quality-filtered and assembled into contigs and scaffolds using SPAdes 3.9.0 (St. Petersburg State University, Russia) [24]. Prediction of the genes present in phage genomes was carried out using Glimmer [25] in the RAST annotation pipeline [26]. Automatic annotations were manually curated using BLASTp searches against the NCBI non-redundant protein database and NCBI Refseq viral database, with the cutoff level set to an *e* value < 10^−4^. Prediction of transmembrane helices was conducted using the TMHMM Server ver. 2.0 [27, 28]. The terminal repeats region of SaGU1 were identified by Bowtie2 then visualized in Geneious Prime 2020.2.5 (https://www.geneious.com) [29].

The phylogenetic tree was generated based on the 17 *Staphylococcus* phage genomes with PhyML [30]. Bootstrap confidence values (100 resamplings) are as indicated on the internal branches [31].

### Nucleotide Sequence Accession Number

The complete SaGU1 genome data has been deposited in the NCBI database under accession number LC574321.

### Statistical Analysis

TEM analysis, one-step growth assay, and the stability analysis data were presented as the mean ± standard deviation (SD) and analyzed using GraphPad Prism version 8.4.3 (471) (GraphPad Software, USA).

## Results and Discussion

### Isolation of Phage SaGU1 Infecting *S. aureus* Clinical Isolates

We obtained sewage samples from the northern plant of the Water and Sewage Division of Gifu City, Gifu, Japan, to screen for phages. Two clinical strains of *S. aureus* (1056-1 and 158-F1) previously isolated from patients with AD were used as host bacteria to screen for the phages. In the screening process, we isolated five and three independent phages from lawns of the strains 1056-1 and 158-F1, respectively. We extracted the genomic DNA from these eight phages to determine their whole genome sequences. All of these isolates showed 100% identical genome sequences; therefore, we selected a single phage infecting 1056-1 as the representative and named it SaGU1.

### Characterization of the SaGU1 Genome

**Fig. 1:**
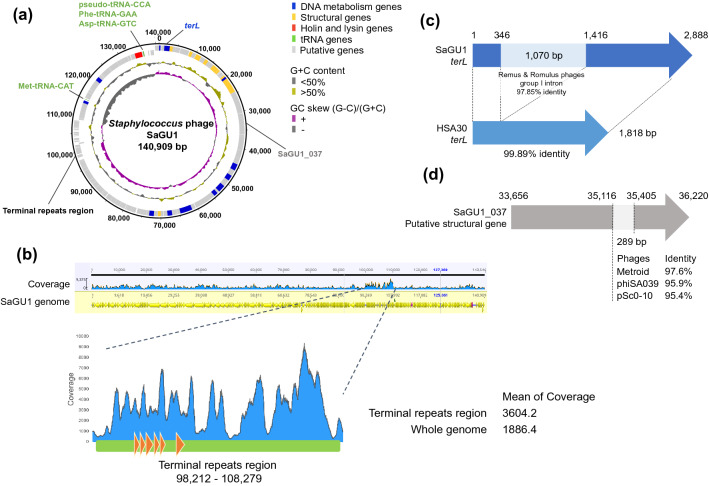
Genomic structure of the phage SaGU1. **a** Circular representation of the SaGU1 genome. Concentric rings denote the following features (from the outer to inner rings): nucleotide positions are forward strand (outer) and reverse strand (inner); the predicted genes are DNA metabolism genes (blue), structural genes (yellow), holin and lysin genes (red), tRNA (green), and putative genes (light gray); G + C content is < 50% (gray), > 50% (gold); GC skew is (G − C)/(G + C) (gray,−; purple, +). **b** Reads mapped onto SaGU1 genome sequence. Predicted terminal repeats region (green) and terminal-repeat encoded proteins (orange) **c** Structure of SaGU1 *terL*. Pale blue line represents the region with high sequence similarity to the group I intron of *Staphylococcus* phages Remus and Romulus. **d** Structure of the SaGU1_037 putative structural gene. Pale gray line represents the region with high sequence similarity to sequences of *Staphylococcus* phages Metroid, phiSA039, and pSc0-10

SaGU1 is a Class III *Staphylococcus* phage with a 140,909 bp genome and an overall GC content of 30.2% (Fig. 1a) [32]. The terminal repeats region of SaGU1 was identified in the 98,212–108,279 nucleotide region (Fig. 1b) [33]. The SaGU1 genome encodes a total of 225 predicted genes and four transfer RNAs (tRNAs). The coding density of the genome was 91%, leaving a very small intergenic region. All predicted gene products were searched against the Refseq protein database; however, only 70 coding sequences (CDSs) had previously assigned functions (Supplementary Table S1).

Topologically, the genome of SaGU1 can be divided into two unequal regions; majority of predicted genes were located on the forward strand, whereas all tRNA genes were on the reverse strand. The tRNA genes were separated into two locations: Met-tRNA-CAT and pseudo-tRNA-CCA, Phe-tRNA-GAA, and Asp-tRNA-GTC (Fig. 1a). This arrangement was conserved in other similar *Staphylococcus* phages. The presence of tRNA in phage genomes is not uncommon, since it has been hypothesized that viral tRNA compensates for the difference in codon usage bias between a phage and its bacterial host, and that the tRNAs correspond to codons that may be inefficiently translated by the host translational machinery [34].

SaGU1 genome was almost identical to that of the *S. aureus* phage HSA30 (MG557618); however, it contained two unique regions. The first region was a locus from 1 to 2888 (Fig. 1c, Table S1). It showed an inserted sequence that spliced the *terL* gene into two segments (SaGU1_1 and SaGU1_3) and was 99.89% identical to the group I intron of Class III *Staphylococcus* phages, Remus (NC_022090), and Romulus (NC_020877) [35]. Introns have been previously reported in various phage genomes [36, 37]. For instance, the intron of certain Twort-like phages has the ability of self-splicing from RNA transcripts [37, 38]; thus, SaGU1_1 and SaGU1_3 are likely self-spliced to express the functional TerL [35, 39]. In addition, based on the BLASTn search for the NT database, the intron in the SaGU1 genome was present in five other genomes of Class III *Staphylococcus* phages; namely, Remus, Romulus, MCE-2014 (NC_025416), StAP1 (KX532239), and phiIPLA-RODI (NC_028765) [35, 40, 41]. The second unique region was a locus from 35,116 to 35,405 in SaGU1_037 (Fig. 1d), which exhibited 98%, 96%, and 95% nucleotide similarity with a region on the genomes of *Staphylococcus* phage Metroid (MT411892), phiSA039 (AP018375), and pSc0-10 (KX011028), respectively. These mosaicisms indicate that horizontal gene transfer is common within this phage group, as mentioned in earlier studies [32, 42–44].

**Fig. 2:**
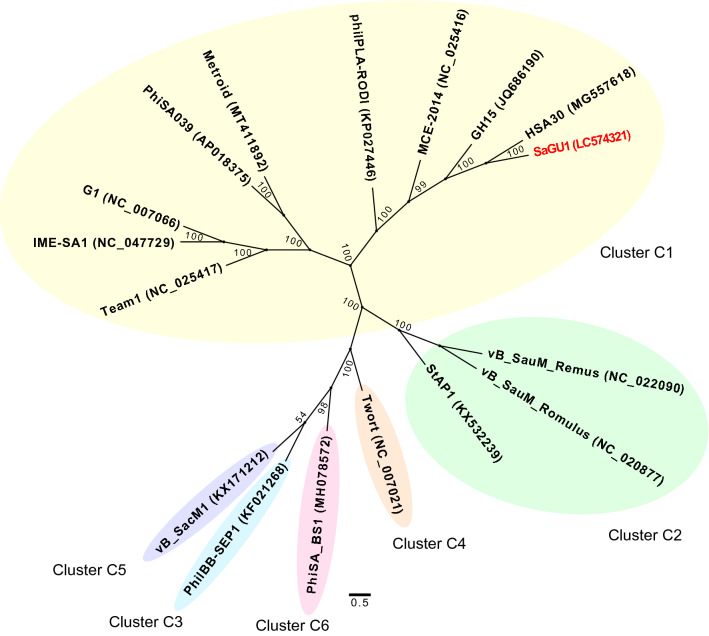
Phylogenetic relationships between SaGU1 and members of the *Staphylococcus* phage Cluster C, based on the whole genome sequencing. A maximum likelihood tree was constructed based on the 6823 nucleotide sites. Bootstrap confidence values (100 re-samplings) are indicated on the internal branches

Phylogenetic analysis was then performed based on the whole genome sequences of SaGU1 and other *Staphylococcus* phages from Cluster C (Fig. 2) [45]. SaGU1 was observed to cluster specifically with the phages of subcluster C1, which generally have a broad host range and therapeutic potential [46].

The gene products with predicted functions encoded modules for virion structure, nucleotide replication and metabolism, and lysis. The structural modules were primarily located between bases 822 and 44,232. However, two genes that encode major tail proteins (SaGU1_71 and SaGU1_72) were located approximately 30 kb upstream from other structural genes, similar to members of kayviruses, such as GH15, MCE-2014, and phILA-RODI [40, 41, 47]. Moreover, the SaGU1 genome possessed genes encoding structural proteins typically present in members of *Myoviridae*, including tail sheath protein (SaGU1_20), tail tube protein (SaGU1_21), and baseplate protein (SaGU1_36) which controls its contractile tails [48].

The lysis module of SaGU1 consisted of two adjacent genes, lysin (SaGU1_216) and holin (SaGU1_217). Based on the in silico prediction, the latter was a member of class II holins, which contain two transmembrane helical domains, with both the N- and C-termini present in the cytoplasm [49]. This module was located downstream of the possible pseudo-tRNA, Phe-tRNA, and Asp-tRNA sequences. As there were no lysogeny-related genes detected in the genome, phage SaGU1 likely depends on the lytic cycle to replicate.

### Morphology of SaGU1

**Fig. 3:**
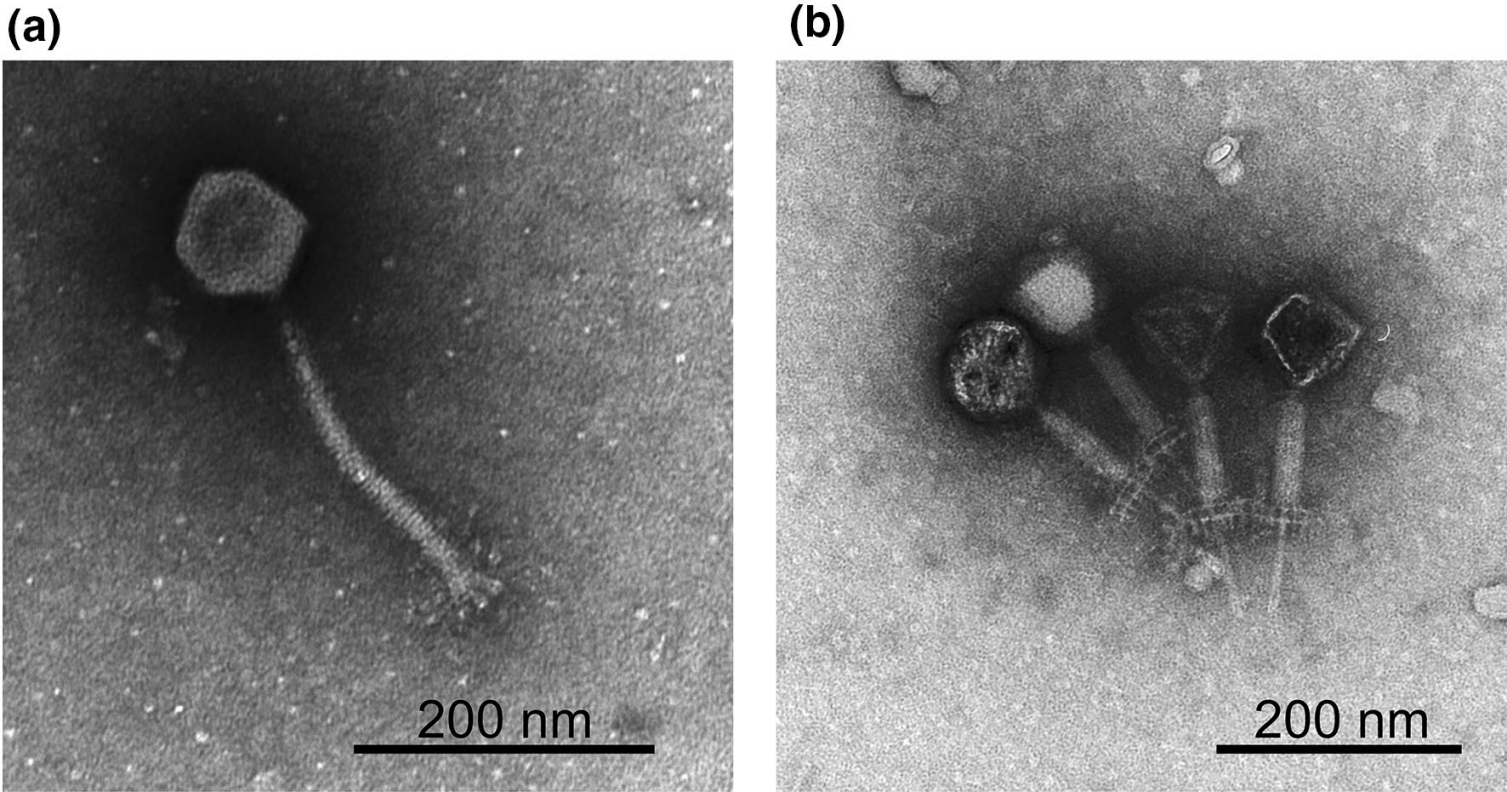
The electron micrographs of phage SaGU1. Pictures were taken by TEM. **a** SaGU1 normal particle. **b** SaGU1 particle with the tail contracted. A characteristic feature of the *Myoviridae* family involves the contraction of the tail sheath, and protrusion of the tail tube from the tip of the tail. A particle with a black head is an empty particle with no DNA. Scale bar, 200 nm

TEM analysis confirmed that SaGU1 possessed an icosahedral head with a diameter of 86.7 ± 5.0 nm (*n* = 3), and a contractile tail with a length of 222.7 ± 1.9 nm (*n* = 3) and a width of 19.3 ± 0.7 nm (*n* = 3) (Fig. 3a). The myovirus tail is concentric with a tail tube inside a tail sheath. The contracted tail sheath is 95.0 ± 5.0 nm (*n* = 3) in length, which is approximately 42.7% of the length before contraction, and a 72.5 ± 2.5 nm (*n* = 3) tail tube protrudes from under the base plate (Fig. 3b). SaGU1 contains a double base plate (Fig. 3b) and no tail fibers, but globular structures at the tail tip (Fig. 3a). A particle appearing with a black head is empty with no DNA (Fig. 3b). When a myovirus infects a host bacterium, the tail contracts and the DNA in the head is ejected. Moreover, the shape of the whole virus resembled that of other staphylococcal phages, Team1 (NC_025417) [50] and Remus [35]. These morphological characteristics indicate that the SaGU1 phage belongs to the genus Twort-like phages of the family *Myoviridae* [51, 52].

### Life Cycle of SaGU1

**Fig. 4:**
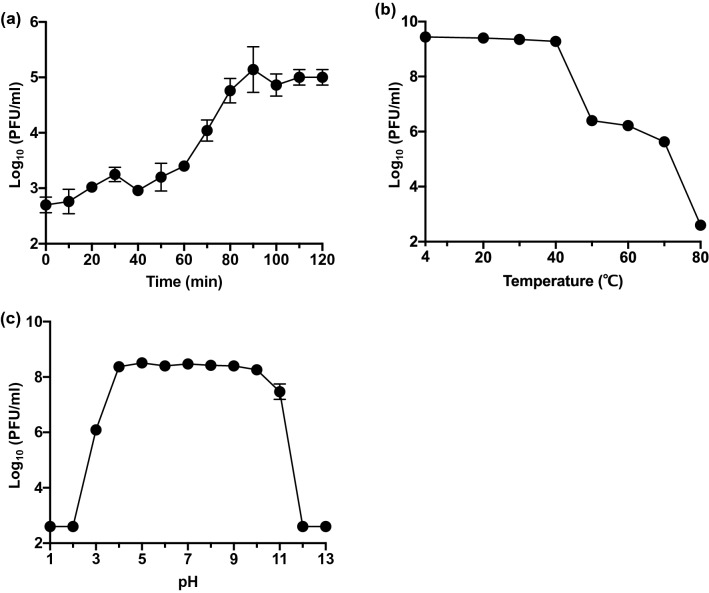
Characterization of phage SaGU1. **a** One-step growth curve of SaGU1 at 37 °C. **b** Effect of temperature on the lytic activity of SaGU1. **c** Effect of pH on the lytic activity of SaGU1. The data are presented as the mean ± standard deviation of at least three independent experiments. Small error bars are obscured by symbols. The detection limit was 4.0 × 10^2^ PFU/mL

An one-step growth experiment was performed to analyze the life cycle of SaGU1 (Fig. 4a). The latent phase was found to be 40 min, followed by a 50 min growth phase. A growth plateau was reached within 90 min. The burst size of SaGU1 was calculated as 117 ± 24 PFU/cell. The life cycle of SaGU1 was comparable to those of other *Staphylococcus* phages in the *Myoviridae* family, Stau2 (NC_030933) (100 PFU/cell), and IME-SA1 (NC_047729) (80 PFU/cell) [53, 54].

### Biophysical Stability of SaGU1

Considering that stable phages are required for phage therapy [55], we examined whether SaGU1 is stable at various temperatures and pH values. Thermostability tests showed that SaGU1 was stable between 4 and 40 °C, with a gradual decrease observed between 50 and 70 °C, and complete inactivation achieved at 80 °C (Fig. 4b). The effects of high and low pH on the stability of SaGU1 were also examined (Fig. 4c). SaGU1 showed stable lytic activity following incubation at conditions between pH 4 and pH 10, whereas it lost significant lytic activity after incubation at conditions below pH 2 and above pH 12. The human skin has a pH of approximately 4.1 to 5.8, with a slightly higher pH in the AD patients (approximately pH 5.5); whereas, a pH of 7.5 is optimal for *S. aureus* growth [56, 57]. These results show that SaGU1 application will be stable on the skin of patients with AD, making it potentially useful for phage therapy.

### Host Specificity of SaGU1

The host specificity of SaGU1 was examined using *Staphylococcus* strains listed in Table 1. The EOP assay results showed that SaGU1 infected 14 of the 16 *S. aureus* strains (EOP values ≥ 0.3). The results of MLST analysis indicated that SaGU1 infected *S. aureus* strains belonging to diverse genomic lineages of sequence type (ST) 4, ST5, ST8, ST6, ST15, and ST398 (*S. aureus* 1056-1 and 158-F1 used for the screening of SaGU1, belonged to ST8 and ST4, respectively), suggesting that SaGU1 infects a broad range of *S. aureus*. Notably, SaGU1 infected the strain GTC01187 (ST5, CC5) that shows resistance to multiple antibiotics, including methicillin. However, SaGU1 did not infect *S. aureus* SA-8 and GTC01196, which belonged to ST25 and ST239, respectively.

We additionally obtained a total of seven known clinical isolates of *S. epidermidis* (two strains from healthy people and five strains from patients with AD), belonging to different genomic lineages based on the MLST analysis, to assess whether SaGU1 infects *S. epidermidis*. The results of EOP assays showed that SaGU1 did not infect any of the *S. epidermidis* strains (EOP < 10^−8^). In addition, SaGU1 did not infect any other Gram-positive or Gram-negative bacteria (Table 1).

Taken together, these data indicate that SaGU1 is a staphylococcal phage that specifically infects *S. aureus* strains of different STs but not *S. epidermidis*. It may, therefore, by useful to establish the host determinants of SaGU1 in future studies.

## Conclusion

A novel *Staphylococcus* phage SaGU1, which was stable under specific physiological and acidic conditions*,* was identified and its complete genomic sequence was determined in this study. Given that SaGU1 can specifically infect *S. aureus* from patients with AD, but not *S. epidermidis*, it may be a strong candidate for developing phage therapy to treat AD.

## Supplementary Information

Below is the link to the electronic supplementary material.

Supplementary file1 (XLSX 20 kb)

## Data Availability

The complete genome data of SaGU1 has been deposited in the NCBI database under accession number LC574321.
